# The cause of redetachment after vitrectomy with air tamponade for a cohort of 1715 patients with retinal detachment: an analysis of retinal breaks reopening

**DOI:** 10.1186/s40662-022-00325-y

**Published:** 2023-02-03

**Authors:** Chuandi Zhou, Chufeng Gu, Bo Li, Yujie Wang, Yanan Hu, Xinping She, Ya Shi, Mingming Ma, Tao Sun, Qinghua Qiu, Ying Fan, Fenge Chen, Hong Wang, Kun Liu, Xiaodong Sun, Xun Xu, Zhi Zheng

**Affiliations:** 1 Department of Ophthalmology, Shanghai General Hospital, Shanghai Jiao Tong University School of Medicine, No. 100 Haining Road, Hongkou District, Shanghai, China; 2 National Clinical Research Center for Eye Diseases, No. 100 Haining Road, Hongkou District, Shanghai, China; 3 Shanghai Key Laboratory of Ocular Fundus Diseases, No. 100 Haining Road, Hongkou District, Shanghai, China; 4 Shanghai Engineering Center for Visual Science and Photomedicine, No. 100 Haining Road, Hongkou District, Shanghai, China; 5 Shanghai Engineering Center for Precise Diagnosis and Treatment of Eye Diseases, No. 100 Haining Road, Hongkou District, Shanghai, China

**Keywords:** Retinal redetachment, Air tamponade, Vitrectomy, Retinal breaks reopening, Postoperative restricted activities

## Abstract

**Background:**

To investigate the prevalence and predictors of retinal breaks reopening after vitrectomy with air tamponade in rhegmatogenous retinal detachment (RRD).

**Methods:**

A retrospective cohort study was conducted in Shanghai General Hospital. Chart review was performed among 1715 patients with primary RRD who received pars plana vitrectomy (PPV) with air tamponade as initial management. Patients were followed up for recurrence. The clinical features of the eyes with retinal breaks reopening were recorded. Logistic regression was constructed to investigate the predictors for breaks reopening.

**Results:**

A total of 137 (7.99%) patients had recurrent retinal detachment after PPV with air tamponade. The causes of surgery failure included new or missed retinal breaks (48.9%), reopening of original tears (43.8%) and proliferative vitreoretinopathy (7.3%). The median time to recurrence for the patients with breaks reopening was 18.0 days. Multivariate logistic regression indicated that the presence of retinal break(s) ≥ 1.5 disc diameters (DD) (odds ratio [OR]: 2.68, 95% confidence interval [CI]: 11.04–6.92, *P* = 0.041), and shorter period for restricted activities (OR: 0.94, 95% CI: 0.89–0.99, *P* = 0.020) were the independent predictors for breaks reopening.

**Conclusions:**

Breaks reopening is an important cause for retinal redetachment after PPV with air tamponade in primary RRD. The first 2–4 weeks after surgery is the “risk period” for breaks reopening. Special attention should be paid for patients with retinal break(s) ≥ 1.5 DD. A prolonged period for restricted activities is recommended.

**Supplementary Information:**

The online version contains supplementary material available at 10.1186/s40662-022-00325-y.

## Background

In the modern era, pars plana vitrectomy (PPV) has become the most common surgical modality for the management of rhegmatogenous retinal detachment (RRD). Intraocular tamponades such as gas and silicone oil are injected at the end of vitrectomy to serve as a barricade to prevent intravitreal fluid entry through retinal breaks and form chorioretinal adhesion. Generally, PPV combined with air tamponade is an important surgical modality reserved for uncomplicated RRDs. Room air is a widely used intraocular tamponade for its faster visual recovery, low cost and lower chances of elevated intraocular pressure (IOP), cataract progression, and proliferative vitreoretinopathy (PVR), when compared with expansile gas [1–3]. In previous years, air tamponade has been more widely used than ever before for the limited access to expansile gas in China. According to previous reports, the single-operation success rate of PPV with air tamponade for RRDs varied from 79.0% to 100% [1, 4–14]. New or missed retinal breaks, reopening of original tears and PVR are the main causes of failure [1, 6, 7, 10–17]. The conditions of new or missed retinal breaks and PVR have been extensively investigated in previous studies [1, 6, 7, 12, 17]. However, to our knowledge, the literature regarding breaks reopening is almost scant.

We conducted a large retrospective cohort study of 1715 patients with primary RRDs treated by PPV with air tamponade to analyze the causes of surgery failure. Of note, breaks reopening accounted for a substantial proportion of recurrence after air tamponade. In this report, we focused on this subgroup of patients with regard to their clinical characteristics to explore the possible correlates of retinal breaks reopening.

## Methods

### Patients

Chart review was performed among patients with primary RRD initially treated by PPV combined with air tamponade. All patients were referred to Shanghai General Hospital from March 2012 to August 2020. The diagnosis of RRD was based on the clinical signs of detached retinas and concurrent retinal breaks, which were also confirmed by B-ultrasonography. The exclusion criteria were: (1) PVR worse than grade C1; (2) giant retinal tears (≥ 1 quadrant); (3) macular hole; (4) history of vitreoretinal surgery; (5) uncapable of maintaining postoperative prone position; (6) follow-up period < 12 months; (7) RRD related to ocular trauma or endophthalmitis; and (8) incomplete data collection for major parameters.

### Data collection

This study adhered to the tenets of the Declaration of Helsinki and was approved by the Shanghai General Hospital research ethics committee. We contacted all patients who met the inclusion criteria and explained the purpose of the study, and they participated in this study voluntarily without any additional compensation. Oral informed consent was obtained from each patient.

Comprehensive ophthalmic examinations were performed pre- and postoperatively, which included: (1) slit-lamp examination with direct and indirect ophthalmoscopy; (2) best-corrected visual acuity (BCVA) using the Snellen visual acuity chart; (3) IOP examined by a non-contact tonometer; and (4) ocular B-ultrasonography.

Age, gender, duration of symptoms, refractive status, the number, type and diameter of retinal breaks, the presence of inferior retinal breaks in detached retina [18], number of quadrants with retinal detachment (RD), grade of PVR, preoperative and postoperative BCVA, lens status, baseline macula status examined by optical coherence tomography, the presence of posterior vitreous detachment (PVD), the method to achieve retinopexy, salvage surgical modality and time to initial recurrence for relapsed cases were collected by chart review. The number of patients having retinal break(s) ≥ 1.5 DD was documented. At the follow-up, each patient was questioned about the period for prone position and restricted activities.

### Surgical procedures

A 23- or 25-gauge three-port PPV was performed using the Alcon Constellation system (Alcon Laboratories, Inc., Fort Worth, TX, USA). After central and peripheral vitreous removal, 360° vitreous base shaving was performed up to ora serrata with scleral indentation. Vitreous traction at the retinal tears was released. A complete fluid-air exchange was performed, and subretinal fluid (SRF) was aspirated through a flute needle. If the retina was highly elevated with extensive area involved, perfluorodecalin was used to flatten the retina first. Most patients underwent endophotocoagulation to achieve retinopexy. However, if the retina breaks were in the peripheral area, transscleral cryopexy was applied. A gentle pressure was maintained on the sclerotomy with a cotton-tip applicator for at least 2 min until the incision site was definitely closed. The IOP was controlled to around 24 mmHg, slightly higher than the normal level, thus avoiding postoperative hemorrhage. All patients were instructed to maintain a proper head positioning to enable the air to tamponade the retinal breaks for 1 week. They were also asked to have restricted activities for 1 month and visited the operating surgeon’s clinic at 1 week, 2 weeks, 1 month, and then bimonthly for at least 12 months. During the follow-up, all patients were interviewed about their own assessments of daily physical activity which were classified as inactive (less than light labor), active (light or moderate labor), or very active (heavy labor) [19]. Restricted activity was defined as being refrained from active or very active physical activity and avoiding frequent and sudden head movement. Anatomical success was defined as the complete disappearance of SRF and flattening of the entire circumference of the retinal breaks.

### Statistical analysis

The computations were analyzed with SPSS 21.0 (IBM Corp, New York, NY, USA). Counting fingers, hand motion and light perception were assigned the logMAR units of 2.1, 2.4 and 2.7, respectively [20]. Frequency (percentage) and median (interquartile range [IQR]) were reported for the description of categorical variables and continuous variables, respectively. Demographic and clinical parameters of patients with and without breaks reopening were compared using either Student’s t-test (continuous factors with normal distribution), nonparametric Mann-Whitney U test (continuous factors with skewed distribution) or the Chi-squared test (categorical factors, or Fisher’s exact test, if appropriate). Multivariate logistic regression analyses were used to determine the predictors for reopening of retinal breaks after controlling for potential confounders. Odds ratio (OR) with its 95% confidence interval (CI) were reported. All the tests were two sided, and a *P* value of less than 0.05 was considered statistically significant.

## Results

Among the 1715 patients with primary RRDs treated by PPV with air tamponade, 137 (8.0%) patients had recurrent retinal detachment. The time to recurrence ranged from 5.0 to 834.0 days, with a median period of 20.0 days. The main causes of surgery failure included new or missed retinal breaks (48.9%), reopening of original tears (43.8%) and PVR (7.3%).

The demographic and preoperative clinical characteristics of patients with and without breaks reopening are tabulated in Table 1. No statistical significance was detected between the two groups with respect to sex, age, duration of symptoms, number of retinal breaks, the presence of inferior retinal breaks in detached retina, PVD, high myopia, retinal degeneration, type of retinal breaks, number of quadrants involved, grade of PVR, macular status, baseline logMAR BCVA, lens status, perfluorodecalin use and the method to achieve retinopexy. Of note, significantly larger proportion of patients had retinal break(s) ≥ 1.5 disc diameters (DD) in the those with reopening eyes (43.3 vs. 24.7%, *P* = 0.021).

**Table 1:** Demographic and baseline clinical characteristics for patients with and without breaks reopening after pars plana vitrectomy with air tamponade

Variables	Total (n = 137)	Reopening of retinal breaks (+) (n = 60)	Reopening of retinal breaks (−) (n = 77)	*P*
Sex				0.377
Male	88 (64.2)	41 (68.3)	47 (61.0)	
Female	49 (35.8)	19 (31.7)	30 (39.0)	
Age (years)	54.0 (45.0–60.0)	52.0 (41.3–63.8)	54.0 (45.5–59.5)	0.841
Duration of symptoms (days)	7.0 (5.0–14.0)	11.5 (5.0–16.5)	7.0 (4.0–14.0)	0.124
Number of retinal breaks	1.0 (1.0–2.0)	1.0 (1.0–2.0)	1.0 (1.0–3.0)	0.700
With inferior retinal breaks in detached retina	49 (35.8)	21 (35.0)	28 (36.4)	0.869
With retinal break(s) ≥ 1.5 DD	45 (32.8)	26 (43.3)	19 (24.7)	0.021*
Type of retinal breaks				0.487
Horse-shoe tear	80 (58.4)	38 (63.3)	42 (54.5)	
Atrophy hole	18 (13.1)	8 (13.3)	10 (13.0)	
Combined	39 (28.5)	14 (23.3)	25 (32.5)	
Number of quadrants involved				0.490
1	38 (27.7)	14 (23.3)	24 (31.2)	
2	53 (38.7)	22 (36.7)	31 (40.3)	
3	34 (24.8)	17 (28.3)	17 (22.1)	
4	12 (8.8)	7 (11.7)	5 (6.5)	
PVR				0.534
A	15 (10.9)	6 (10.0)	9 (11.7)	
B	114 (83.2)	49 (81.7)	65 (84.4)	
C1	8 (5.8)	5 (8.3)	3 (3.9)	
Macular status				0.340
On	40 (29.2)	15 (25.0)	25 (32.5)	
Off	97 (70.8)	45 (75.0)	52 (67.5)	
With myopia	55 (40.4)	22 (37.3)	33 (42.9)	0.512
With PVD	70 (58.8)	32 (62.7)	38 (55.9)	0.452
With retinal degeneration	85 (62.0)	36 (60.0)	49 (63.6)	0.663
Baseline BCVA (logMAR)	2.0 (0.7–2.1)	2.1 (0.7–2.1)	2.0 (0.7–2.1)	0.612
Preoperative lens status				0.672
Phakic	122 (89.1)	54 (90.0)	68 (88.3)	
Pseudophakic	14 (10.2)	6 (10.0)	8 (10.4)	
Aphakic	1 (0.7)	0 (0)	1 (1.3)	
Perfluorodecalin use	21 (15.3)	11 (18.3)	10 (13.0)	0.389
Method to achieve retinopexy				0.113
Laser photocoagulation	98 (71.5)	38 (63.3)	60 (77.9)	
Cryotherapy	22 (16.1)	12 (20.0)	10 (13.0)	
Combined	17 (12.4)	10 (16.7)	7 (9.1)	

Data presented as median (interquartile range) or n (%)

*DD* = disc diameter; *PVR* = proliferative vitreoretinopathy; *PVD* = posterior vitreous detachment; *logMAR* = logarithm of the minimum angle of resolution; *BCVA* = best-corrected visual acuity

*Indicates statistically significant

The postoperative clinical data are summarized in Table 2. The median time to initial recurrence caused by breaks reopening was 18.0 days, which was remarkably shorter than those without this sign (28.0 days, *P* = 0.003). Among 60 patients with retinal breaks reopening, 20 (33%) patients relapsed within 2 weeks postoperatively, and 30 (50%) recurred at 3–4 weeks. Moreover, the period for restricted activities for patients with breaks reopening was also much shorter (13.5 vs. 17.0 days, *P* = 0.020). For relapsed cases, most of them received repeated PPV with silicone oil tamponade (102, 74.5%), and the residual (35, 25.5%) underwent repeated PPV with air tamponade as the salvage treatment. The retinal reattachment rate after salvage treatments was 96.4%. Of note, no further difference was identified between the two groups with respect to prone position period, final logMAR BCVA, lens status, types of salvage treatments and final retinal reattachment rate.

**Table 2:** Postoperative clinical characteristics for patients with recurrent retinal detachment after pars plana vitrectomy with air tamponade

	Total (n = 137)	Reopening of retinal breaks (+) (n = 60)	Reopening of retinal breaks (−) (n = 77)	*P*
Time to recurrence (days)	20.0 (12.5–41.0)	18.0 (12.0–25.0)	28.0 (13.0–58.5)	0.003*
Prone position period (days)	6.0 (5.0–7.0)	6.0 (5.0–8.0)	6.0 (5.0–7.0)	0.594
Period for restricted activities (days)	15.0 (10.0–22.0)	13.5 (8.3–20.0)	17.0 (12.5–24.0)	0.020*
BCVA at the last visit (logMAR)	0.5 (0.4–1.0)	0.5 (0.4–1.0)	0.5 (0.4–1.0)	0.510
Lens status at the last follow-up				0.370
Phakic	91 (66.9)	42 (71.2)	49 (63.6)	
Pseudophakic	40 (29.4)	14 (23.7)	26 (33.8)	
Aphakic	5 (3.7)	3 (5.1)	2 (2.6)	
Salvage treatment				0.509
PPV with air tamponade	35 (25.5)	17 (28.3)	18 (23.4)	
PPV with silicone oil tamponade	102 (74.5)	43 (71.7)	59 (76.6)	
With final retinal reattachment	132 (96.4)	57 (95.0)	75 (97.4)	0.653

Data presented as median (interquartile range) or n (%)

*BCVA* = best-corrected visual acuity; *logMAR* = logarithm of the minimum angle of resolution; *PPV* = pars plana vitrectomy

^*^ Indicates statistically significant

**Fig. 1:**
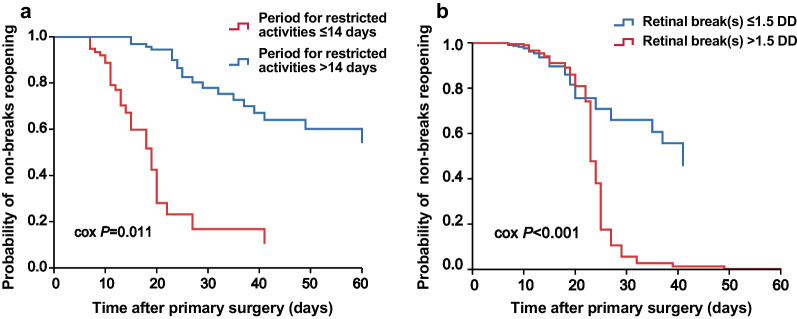
Restricted activity peroid ≤ 14 days and retinal break(s) ≥ 1.5 DD are predictive of local recurrence. **a** Compared with those of restricted activity days ≤ 14 days, patients with restricted activity days > 14 days had a lower cumulative incidence of local recurrence (*P* = 0.011). **b** Compared with patients with retinal break(s) ≥ 1.5 disc diameter (DD), those of break(s) < 1.5 DD had a lower cumulative incidence of local recurrence (*P* < 0.001)

Further analysis with multivariate logistic regression indicated that the presence of retinal break(s) ≥ 1.5 DD (OR: 2.68, 95% CI: 1.04–6.92, *P* = 0.041) and shorter period for restricted activities (OR: 0.94, 95% CI: 0.89–0.99, *P* = 0.020) were independent predictors for breaks reopening after adjusting for the potential correlates of surgery failure (Table 3). Cumulative probability of retinal breaks reopening during the follow-up for the patients stratified by the presence of retinal break(s) ≥ 1.5 DD and different periods for restricted activities are displayed in Fig. 1. Of note, we excluded the patients with delayed redetachment (> 100 days postoperatively) to disperse the events more clearly plotted by follow-up period.

**Table 3:** Multivariate logistic regression of risk factors for reopening of retinal breaks

	Odds ratio (95% CI)	*P*
Sex (female vs. male)	0.46 (0.18–1.22)	0.119
Age (years)	0.99 (0.96–1.03)	0.690
Duration of symptoms (days)	1.00 (0.99–1.01)	0.660
Number of retinal breaks	1.10 (0.64–1.91)	0.730
With inferior retinal breaks in detached retina (yes vs. no)	1.29 (0.51–3.24)	0.593
With retinal break(s) ≥ 1.5 DD (yes vs. no)	2.68 (1.04–6.92)	0.041*
Number of quadrants involved	1.25 (0.75–2.08)	0.389
Method to achieve retinopexy		
Laser photocoagulation	1 [Reference]	
Cryotherapy	2.64 (0.83–8.43)	0.102
Combined	2.08 (0.44–9.96)	0.359
PVR		
A	1 [Reference]	
B	0.71 (0.17–2.94)	0.636
C1	0.56 (0.03–9.48)	0.685
Macular status (off vs. no)	0.98 (0.37–2.61)	0.966
Posterior vitreous detachment (yes vs. no)	1.03 (0.42–2.55)	0.951
Period for restricted activities (days)	0.94 (0.89–0.99)	0.020*

*DD* = disc diameter; *PVR* = proliferative vitreoretinopathy; *CI* = confidence interval

*Indicates statistically significant

## Discussion

Our study demonstrated that the single-operation success rate of PPV with air tamponade for the 1715 patients with RRDs was 92.0%, which is in tandem with previous studies where the primary reattachment rate varies from 79.0% to 100.0% [1, 4–14]. We analyzed the failure causes and described their distribution. Consistently, the leading cause was new or missed breaks, which accounted for nearly half of the failure cases (48.9%) [1, 6, 7, 10–16]. Breaks reopening comprised the second frequent cause, accounting for 43.8%, followed by PVR (7.3%).

Undetected breaks or formation of new breaks, and progressive PVR after vitrectomy have been well characterized [1, 17]. However, in most reported cases of reopened breaks, no definite cause was allocated. In this study, patients with large retinal break(s) (≥ 1.5 DD) had higher chances of breaks reopening. From our intraoperative observation during salvage vitrectomy, incomplete adhesion of large breaks, and most of the chorioretinal scar failed to develop at the anterior border. This finding is parallel with Tornambe et al. who observed that large tears up to 2.5 clock hours in size was a predictor for failure in pneumatic retinopexy [21].

Importantly, there was a trend where the median duration of symptoms for eyes with reopened break (11.5 days) was longer than those without (7.0 days), but it did not reach statistical significance. This finding is compiled with our clinical notions that long-lasting retinal detachment is a presumed risk factor for surgery failure for a higher grade of preoperative PVR.

From our observation, the median time to recurrence caused by reopening of retinal breaks (18.0 days, IQR: 12.0–25.0 days) was remarkably shorter than those without breaks reopening (28.0 days, IQR: 13.0–58.5 days). Although numerous studies have suggested that adhesion between the neurosensory retina and the retinal pigment epithelium (RPE) developed within 24 h of vitrectomy [22–24]. Postoperatively, with proper prone positioning, air tamponade closed retinal breaks by its surface tension and buoyancy [25]. However, the adhesion formed in the early days may not be sufficiently strong to seal the retinal edge around the tear, indicating that time is required for permanent scar formation [23, 24]. The shear stress at the circumference of breaks leads to its reopening. This stress is maximum with sudden jerking head movements and greatest at the fluid-air interface [26]. Also, with the head movements, residual SRF could shift, especially to the inferior breaks, in such cases, shifted SRF could cause reopening of treated retinal breaks in the early postoperative period. In this study, all the patients were instructed to maintain a proper head positioning for 1 week and have restricted activities for 1 month. However, in the follow-up interview, most of them completed prone positioning but could not adhere to restricted activities for 1 month. The cases with reopened breaks had significantly less time of restricted activities than the those without. As the air tamponade disappeared within the 2 weeks after vitrectomy [27], most of patients return to work at this time for rapid visual rehabilitation with air tamponade. However, among 60 patients with retinal breaks reopening, 33% of them relapsed within 2 weeks postoperatively, and half of them recurred at 3–4 weeks. Therefore, instead of emphasizing the prone positioning, restricted activities should also be considered a critical factor. In agreement with previous findings, Martínez-Castillo et al. yielded favorable success rate for RRD with inferior breaks repaired by PPV with gas tamponade after 24-h facedown position compared with no prone position [13–15]. This series study demonstrated that facedown position did not influence the development of a chorioretinal adhesion in the treated retinal tears. Further, we would not recommend the avoidance of long-acting gas tamponade in all patients, especially for the patients with poor compliance since an intraocular gas bubble may act as a reminder that excess and vigorous head movements should be avoided. An animal study revealed that it was not until 4 months after surgery that the regenerated RPE cells presented with the same morphological characteristics as normal RPE cells. Fluorescein angiography showed no leakage at the original RPE wound by 1 month postoperatively, indicating that the blood-retinal barrier was reconstructed by the regenerated RPE cells [28]. Although most authors did not indicate whether patients maintained restricted activities or not, or how long it lasted, we recommend prolonged period for restricted activities to bring the retina and choroid in contact for sufficient time to produce a firm chorioretinal adhesion to permanently close the break and allow the SRF to be completely cleared by the RPE.

In addition, traction by the remnant vitreous near the edge of retinal tears may reopen sealed breaks. In the salvage vitrectomy, a thin and condensed vitreous was observed at the retinal breaks in most cases. Conformed to previous publications, vitreoretinal traction remains an important cause of breaks reopening [29]. Vitreoretinal surgical expertise is demanded to remove vitreous completely around retinal breaks and in the periphery at the vitreous base. This can be achieved by doing total PPV and 360° vitreous base shaving under prism lens with higher magnification. Triamcinolone was used to visualize the vitreous (Additional file 1). From our observation, a thorough vitreous cortex removal is beneficial for preventing recurrence. Parallel findings were seen in previous studies where the vitreous cortex remnants (VCR) increased the recurrence of retinal detachment due to persistent tangential traction to form new breaks or reopen preexisting breaks [30]. In addition, some authors have suggested that peripheral VCR induced PVR [31], and VCR at the macula may act as a scaffold for fibrocellular proliferation [32]. Therefore, adequate triamcinolone-assisted VCR removal at both posterior pole and midperipheral should be performed to prevent recurrence.

This report analyzed the causes of breaks reopening in an attempt to highlight the important preoperative, operative, and postoperative factors that can contribute to redetachment, and by taking prophylactic measures, the surgical outcome can be improved. During preoperative assessment, it is crucial to examine the detached retina to locate the breaks and zones with vitreoretinal traction, particularly in patients with large breaks. Intraoperatively, a 360° scleral indentation should be performed to identify all breaks and meticulous peripheral vitrectomy is required. A complete fluid-air exchange, definite laser and cryopexy retinopexy also account for the anatomic success. Furthermore, the sclerotomy should be securely closed to avoid hypotony immediately after surgery. Finally, education on postoperative refraining from vigorous exercise for 1 month and regular follow-ups are also critical.

This study should be regarded as an initial exploration of the prevalence and predictors of retinal breaks reopening in primary RRDs managed by PPV with air tamponade. However, several limitations have hindered the interpretation of our findings. First, this study was retrospective in nature. Patient-reported outcomes may have been subjected to recall bias. The exclusion of patients lost to follow-up 12 months after surgery can also underestimate recurrence rate. Nevertheless, the inclusion of a large and heterogeneous patient sample, the use of rigorous statistical methodology and adjustment of various interactions helped to eliminate bias and made our results more convincing.

## Conclusions

Breaks reopening is an important cause of surgery failure for patients with primary RRDs treated by vitrectomy with air tamponade. The first 2–4 weeks after surgery is the “risk period” for breaks reopening. Special attention should be paid for patients with retinal break(s) ≥ 1.5 DD. A prolonged period for restricted activities is recommended to prevent reopening of retinal breaks.

## Supplementary Information

**Additional file 1.** A video showing posterior vitreous detachment induction assisted by triamcinolone.

## Data Availability

All data generated or analyzed during this study are included in this published article.

## Abbreviations

BCVA: Best-corrected visual acuity
CI: Confidence interval
DD: Disc diameters
IOP: Intraocular pressure
OR: Odds ratio
PPV: Pars plana vitrectomy
PVD: Posterior vitreous detachment
PVR: Proliferative vitreoretinopathy
RD: Retinal detachment
RPE: Retinal pigment epithelium
RRD: Rhegmatogenous retinal detachment
SRF: Subretinal fluid
VCR: Vitreous cortex remnants
